# A rise in saliva and urine pH in children with *SCN1A*-related epilepsy: An exploratory prospective controlled study

**DOI:** 10.3389/fneur.2022.982050

**Published:** 2022-09-27

**Authors:** Qian Lu, Yang-Yang Wang, Hui-Min Chen, Qiu-Hong Wang, Xiao-Yan Yang, Li-Ping Zou

**Affiliations:** ^1^Senior Department of Pediatrics, Chinese PLA General Hospital, Beijing, China; ^2^Department of Pediatrics, The First Medical Center of Chinese PLA General Hospital, Beijing, China; ^3^Beijing Institute for Brain Disorders, Center for Brain Disorders Research, Capital Medical University, Beijing, China

**Keywords:** SCN1A, Dravet syndrome, epilepsy, saliva pH, urine pH

## Abstract

**Objective:**

*SCN1A*, encoding the alpha 1 subunit of the sodium channel, is associated with a range of related epilepsy. This study aims to assess saliva and urine pH in children with *SCN1A*-related epilepsy.

**Methods:**

A prospective controlled observational study with a 1:1 ratio was conducted on seven patients with *SCN1A*-related epilepsy and seven healthy children of the same family, gender, and age but without a history of seizures. The pH of saliva and urine was measured by pH test paper. Parents of patients with epilepsy recorded seizures to compare the relationship between pH and seizures.

**Results:**

The fourteen participants were all males, aged 1 to 14 years. Seven patients had different pathogenic *SCN1A* variants. The pH of saliva and urine was monitored for 21–95 days. The pH of saliva and urine was higher in patients with *SCN1A*-related epilepsy than in the healthy group. The urine pH in Dravet syndrome patients was high compared with other epilepsy patients. The urine pH in patients with seizures was higher than that in patients without seizures, which occurred during the study.

**Conclusions:**

The pH of saliva and urine was chronically high in patients with *SCN1A*-related epilepsy, and urine pH was higher in patients with seizures and with Dravet syndrome.

## Introduction

Epilepsy is a common chronic neurological disease and affects about 0.5–1% of children (1). About 50 million people worldwide have epilepsy, of which 25% are children under 15 years of age (2, 3). The etiology of epilepsy is complex and includes genetic, structural, infectious, immune, metabolic, and unknown etiologies (4). Voltage-gated sodium ion channels (VGSCs) are involved in the generation of action potentials in neural cells, and mutations associated with these channel genes are common causes of genetic epilepsies (5).

*SCN1A* locates at 2q24.3 and encodes the α1 subunit of the sodium channel (Nav1.1). Nav1.1 is mainly distributed in neuron cell bodies, dendrites, and the initial segment of axons in the central nervous system (6). *SCN1A*-related diseases have a wide range of clinical phenotypes, which are associated with a variety of epileptic types, such as febrile seizure and Dravet syndrome (7). Sudden and repeated seizures not only affect the health of children but also cause psychological burdens for their parents.

Elevated pH leads to increased neural excitability. An increase in brain pH was observed during seizures in a rat model of febrile seizures (8). Hot water bathing could induce respiratory alkalosis in *Scn1a* mutant rats (9). Inhalation of carbon dioxide rapidly terminates hyperthermia-induced seizures in febrile seizure rats and *Scn1a* mutant rats (8, 9). We previously found that 5% CO_2_ reduced the seizures of the kainic acid rat model by reducing the cortex pH (10).

Seizures cause pH fluctuations in the brain. Functional magnetic resonance imaging is a non-invasive method used to detect brain pH (11). Although this method is sensitive, it cannot continuously monitor brain pH. Blood pH analysis is inconvenient, especially during the COVID-19 pandemic. For children, non-invasive methods such as saliva and urine collection are simple and can be performed daily at home.

Therefore, we aimed to assess the pH of saliva and urine in a series of patients with *SCN1A*-related epilepsy. In this study, the pH of saliva and urine were compared between patients and controls of the same age, gender, and family.

## Methods

### Participants and study design

**Figure 1:**
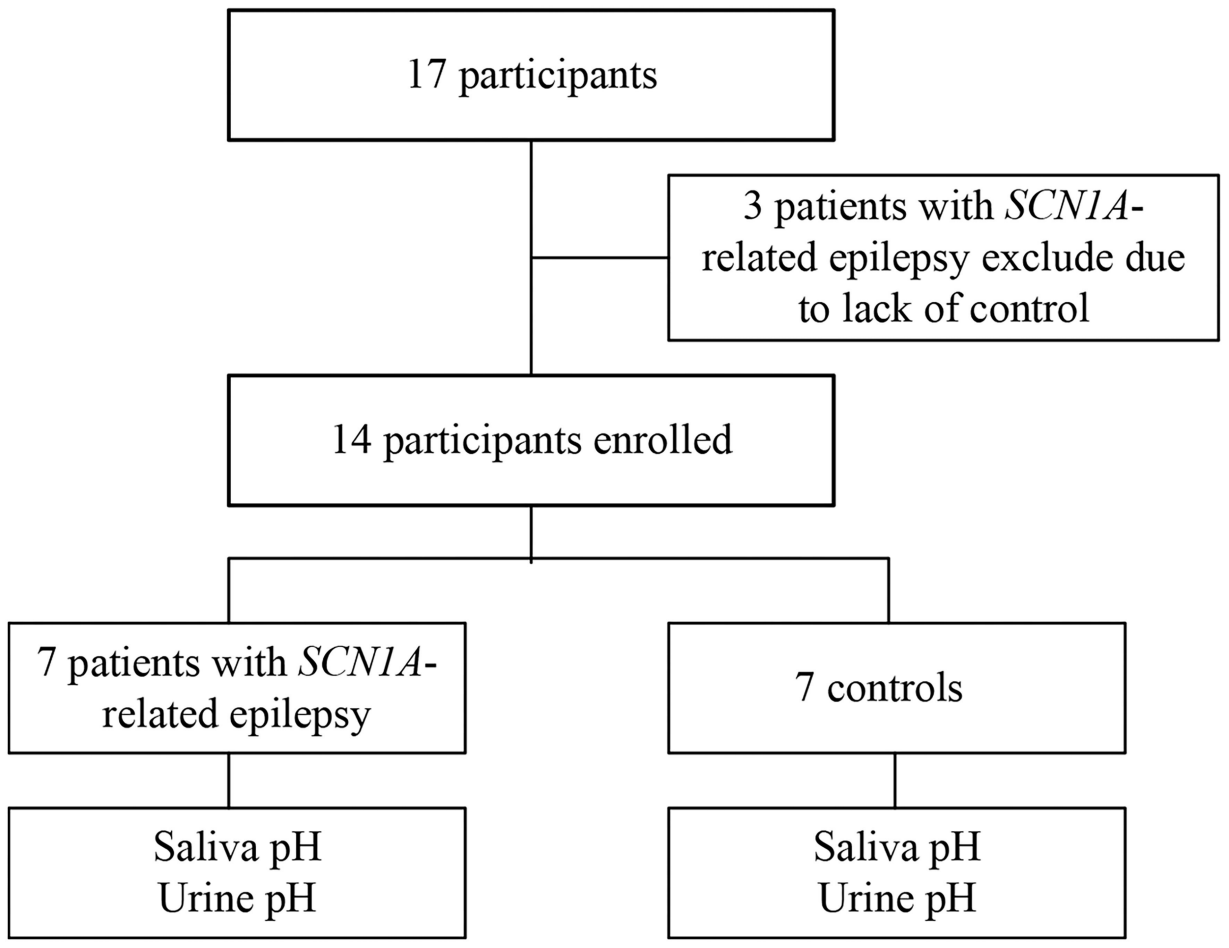
Flow diagram of study participants.

We included ten patients with epilepsy with pathogenic *SCN1A* variants who were treated in the Department of Pediatrics, the First Medical Center of PLA General Hospital from November 2019 to January 2021. Seven healthy children of the same age, gender, and family but without a history of seizures were included in the control group. Three patients with epilepsy were excluded because they could not find suitable healthy children in the control group (Figure 1). We collected the age, gender, home address, birth history, family history, and history of medication of participants. This study obtained informed consent from the guardians and was conducted following the Declaration of Helsinki.

The pH of saliva and urine was measured for all participants by their patients using pH test paper (Geshan OB/440100710745-2007) (Supplementary Figure 1), and the measurement range of pH was 4.50–9.00. Before the beginning of the study, parents had been trained to interpret the color of the pH test paper. Parents must truthfully record pH values. Saliva pH was measured after getting up in the morning without oral activities such as eating and gargling before measurement. The pH test paper was placed in the middle of the tongue. After the test paper was soaked with saliva, it was taken out and compared with the standard color card to record the saliva pH. Urine pH was measured in clean-catch midstream urine in the morning. After the test paper was soaked with urine, it was taken out and compared with the standard color card to record the urine pH.

### Variant detection

Genomic DNA was extracted from the whole blood of patients with epilepsy and their parents. Patients 1, 3, and 6 were subjected to the whole exome-sequencing with an average sequencing depth of 100× and covering upstream and downstream 50 bp. A febrile seizure panel with 189 genes related to febrile seizure was performed on patients 2 and 5. An epilepsy gene panel with 152 genes related to epilepsy was performed on patient 4. Sanger sequencing of *SCN1A* was performed on patient 7. Variants were screened for the various types, genetic patterns, population frequencies, and a list of genes associated with the main phenotypic characteristics of the patients. All parents were validated by Sanger sequencing. The pathogenicity of the variants was evaluated by Mutation Taster and PolyPhen-2.

### Statistical analysis

Non-normally distributed data were presented as median with interquartile. SPSS24.0 was used for statistical analysis. Wilcoxon test was used to assess the difference between the two groups. A *p*-value of < 0.05 indicated statistical significance.

## Results

### Clinical phenotype

Fourteen children were included in this study. Seven children were patients with *SCN1A*-related epilepsy, including two Dravet syndrome patients (patients 1 and 7). Seven healthy children of the same family, age, and gender without a history of seizures were classified as the healthy group (Table 1). The children were boys aged 1 to 14 years. The monitoring time of pH ranged from 21 to 95 days. The onset age of seven patients with epilepsy was <1 year old. Four patients had tonic–clonic seizures, one patient had tonic seizures, one patient had clonic seizures, and one patient had epileptic spasms seizures. Three patients took more than two antiseizure medications. Patients 1, 3, and 7 had seizures during the study.

**Table 1:** Clinical characteristics of the fourteen participants.

**Number**	**Sex**	**Age^#^ (years)**	**Duration of monitoring (days)**	**Onset ages (months)**	**Classification of seizure types**	**Frequency of seizure(/month)**	Treatment^*^
Patient 1	Male	1	90	7	Tonic	3.7	CLB, LEV, ZNS
Patient 2	Male	14	95	8	Clonic	0	VPA
Patient 3	Male	1	88	8	Tonic-clonic	1.7	LEV, VPA
Patient 4	Male	6	30	6	Tonic-clonic	0	LEV, VPA
Patient 5	Male	10	21	5	Tonic-clonic	0	VPA, TPM
Patient 6	Male	1	21	4	Epileptic spasms	0	TPM, VGB, CLB, VPA
Patient 7	Male	9	27	9	Tonic	1	VPA, CLB, TPM
Control 1	Male	1	90	-	-	-	-
Control 2	Male	14	95	-	-	-	-
Control 3	Male	1	88	-	-	-	-
Control 4	Male	6	30	-	-	-	-
Control 5	Male	10	21	-	-	-	-
Control 6	Male	1	21	-	-	-	-
Control 7	Male	9	27	-	-	-	-

# : The age column showed the age of monitoring the pH of the children.

* : CLB, clobazam; LEV, levetiracetam; ZNS, zonisamide; VPA, sodium valproate; TPM, topiramate; VGB, vigabatrin.

### Variant of *SCN1A*

**Figure 2:**
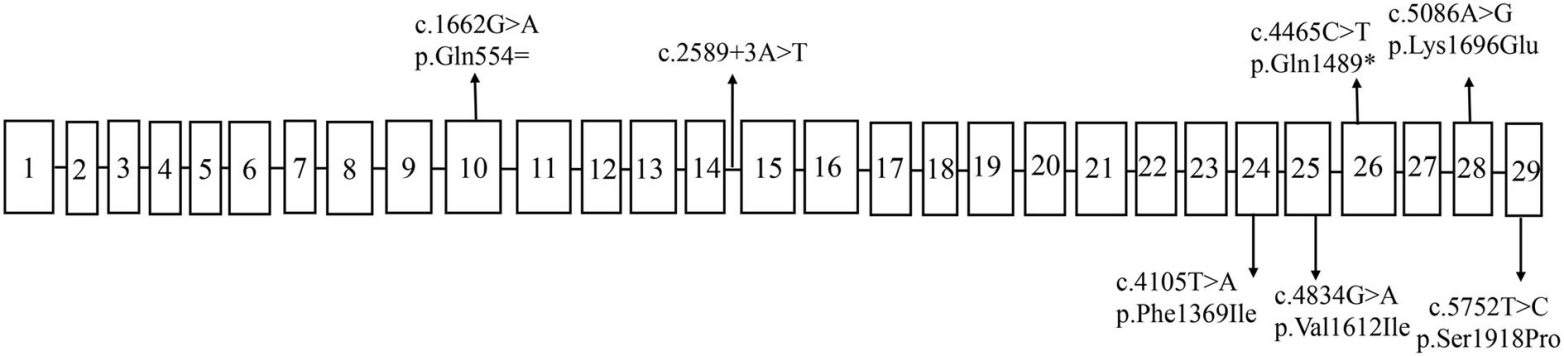
The distribution of *SCN1A* variants sites.

Seven patients with *SCN1A*-related epilepsy had seven different *SCN1A* variants (NM_001165963.4) (Table 2). Figure 2 shows the distribution of *SCN1A* variant sites. Patient 1 (c.4465C>T p.Gln1489^*^) had a *de novo* nonsense variant. Patient 5 (c.1662G>A p.Gln554=) had a *de novo* synonymous variant, which was reported to be associated with epilepsy (12) and was pathogenic in the ClinVar database (https://www.ncbi.nlm.nih.gov/clinvar/variation/206771/). Patient 7 (c.2589 +3A>T) had a *de novo* splicing variant located in intron 14, which was reported to be associated with epilepsy (12) and was also included in the *SCN1A* variants database (http://scn1a.caae.org.cn/scn1a_variant.php). Patient 4 (c.4834G>A p.Val1612Ile) had a missense variant, the same variant as his mother, which was associated with epilepsy in infancy and also inherited from the mother (13). The three other patients had *de novo* missense variants. Variants in patient 2 (c.5086A>G p.Lys1696Glu), patient 3 (c.5752T>C p.Ser1918Pro) and patient 6 (c.4105T>A p.Phe1369Ile) were not reported. The evaluation results of Mutation Taster and Polyphen-2 and the American College of Medical Genetics and Genomics (ACMG) classification of all patients are shown in Table 2.

**Table 2:** Analysis of *SCN1A* variants of seven patients. (NM_001165963.4).

**Number**	**Variant**	**Amino acid alteration**	**Variant type**	**origin**	**Mutation taster**	**Polyphen-2**	**ACMG classification**
Patient 1	c.4465C>T	p.Gln1489^*^	Nonsense variant	De novo	Disease causing	-	Pathogenic
Patient 2	c.5086A>G	p.Lys1696Glu	Missense variant	De novo	Disease causing	Probably damaging (0.997)	Likely pathogenic
Patient 3	c.5752T>C	p.Ser1918Pro	Missense variant	De novo	Disease causing	Probably damaging (1.000)	Likely pathogenic
Patient 4	c.4834G>A	p.Val1612Ile	Missense variant	Maternal	Disease causing	Probably damaging (0.903)	Pathogenic
Patient 5	c.1662G>A	p.Gln554=	Synonymous variant	De novo	Disease causing	-	Pathogenic
Patient 6	c.4105T>A	p.Phe1369Ile	Missense variant	De novo	Disease causing	Probably damaging (0.999)	Likely pathogenic
Patient 7	c.2589+3A>T	-	Splice variant	De novo	-	-	Pathogenic

* Represents code termination.

### A rise in saliva and urine pH in children with *SCN1A*-related epilepsy compared with the healthy group

**Figure 3:**
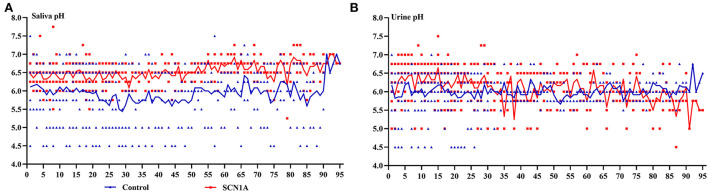
The pH of fourteen participants. **(A)** Shows saliva pH. The saliva pH distribution of patients with *SCN1A*-related epilepsy was higher than that in the healthy group, and the average pH curve was higher than that in the healthy group. **(B)** Shows urine pH, which distribution of patients was higher than that in the healthy group. The horizontal axis is the number of monitoring days, the vertical axis is the pH, and the curve is the average pH. The blue triangle is the pH of the healthy group, and the red square is the pH of patients with *SCN1A*-related epilepsy.

The saliva and urine pH of the participants are shown in Figure 3. The red square represents patients with *SCN1A*-related epilepsy, and the blue triangle represents the control group. The saliva pH of the patients was higher than that in the control group [6.5 (6.25, 6.75) vs. 6.00 (5.50, 6.50), *p* < 0.001]. The urine pH of the patients was higher than that in the control group [6.25 (5.75, 6.50) vs. 6.00 (5.75, 6.25), *p* < 0.001].

### The urine pH in Dravet syndrome patients was high compared with other epilepsy patients

Patients 1 and 7 were diagnosed with Dravet syndrome. The saliva pH of the two Dravet syndrome patients (the mean pH is 6.38) was lower than that of other epilepsy patients (the mean pH is 6.54) (*p* < 0.001). The urine pH of the two Dravet syndrome patients was higher than that of other epilepsy patients [6.50 (6.00, 6.75) vs. 6.00(5.75, 6.50), *p* < 0.001] (Supplementary Figure 2).

### The urine pH in patients with seizures was higher than that in patients without seizures occurred during the study

Patients with *SCN1A*-related epilepsy were divided into two groups according to whether they had seizures during the study. Patients 1, 3, and 7 had seizures during the study. The saliva pH of the three patients who had seizures was higher than that of the healthy group [6.50 (6.40, 6.75) vs. 5.75 (5.00, 6.00), *p* < 0.001]. The urine pH of the three patients who had seizures was higher than that of the healthy control group [6.25 (6.00, 6.75) vs. 6.00 (5.75, 6.25), *p* < 0.001].

We compared the saliva and urine pH in patients who had seizures or not. Patients with seizures had higher urine pH than those without seizures that occurred during the study [6.25 (6.00, 6.75) vs. 6.00 (5.74, 6.50), *p* < 0.001]. There was no difference in saliva pH between the two groups (*p*= 0.114) (Supplementary Figure 3).

## Discussion

The saliva and urine pH were chronically high in this series of patients with *SCN1A*-related epilepsy. There was no study on the saliva and urine pH in patients with *SCN1A*-related epilepsy. However, there are more studies on epilepsy and acidosis (8–10). We previously reported that 5% CO_2_ inhalation can suppress hyperventilation-induced absence seizures in children (14). Acid-sensing ion channel 1a (ASIC1a) is widely expressed in the central nervous system and is sensitive to extracellular pH. Disrupting mouse ASIC1a increased the severity of seizures and CO_2_ inhalation did not terminate seizures, while overexpressing ASIC1a had the opposite effect (15). Acidosis and ASIC1a play an important role in epilepsy (16, 17). Inhalation of CO_2_ can suppress seizures by reducing brain pH and activating ASIC1a. Acidosis activates ASIC1a and inhibits N-methyl-D-Aspartate (NMDA) receptors to reduce seizures (18). Acidosis also terminates seizures by activating ASIC1a and inhibiting interneurons (15). From the studies, we know that acid and base changes have a great impact on epilepsy. Thus, we chose a non-invasive method to measure the pH in children with *SCN1A*-related epilepsy.

Antiseizure medications, such as valproic acid, levetiracetam, and carbonic anhydrases, could decrease brain pH, suggesting that acidosis contributes to antiepileptic effects (19–21). Topiramate is a carbonic anhydrase inhibitor used to treat many types of epilepsy. Studies have found that topiramate can appropriately lower the pH of neuronal cells (22, 23). Topiramate has specific adverse effects, such as hypohidrosis and hyperthermia (24, 25). In epileptic children, the body temperature can reach 38.8°C (26). Hyperthermia is contraindicated for heat-sensitive epilepsy (*SCN1A*-related epilepsy such as Dravet syndrome). However, topiramate is effective in patients with Dravet syndrome, and approximately 35–78% of patients have reduced seizures by more than 50% (27). In our study, topiramate was used in three patients (patients 5, 6, and 7). The saliva pH of the three patients treated with topiramate (the mean pH is 6.43) was lower than that of those (the mean pH is 6.51) not treated with topiramate (*p* = 0.004). Three patients had higher urine pH than those not treated with topiramate [6.75 (6.50, 6.75) vs. 6.50 (6.25, 6.50), *p* < 0.001]. Studies have shown that TPM increases urine pH (28), and so does our patient's urine pH.

Saliva pH was measured with an electrode pH meter in the previous study (29). We used pH test paper to measure pH, which is easy to operate and cheap. And it is highly accepted by parents. Our study investigated the pH levels of saliva and urine in patients with *SCN1A*-related epilepsy, blood pH was not involved in the study. Diets rich in cereal grains and animal protein may decrease pH. A high intake of fruits and vegetables may increase the urine pH (30). Therefore, saliva and urine pH monitoring could guide the diet of epilepsy patients.

The study has limitations. We recruited a small number of patients. Diet will affect the pH of saliva and urine. We selected children from the same family as the control group to reduce the influence of diet. Therefore, we did not strictly control the diet of the participants.

## Conclusion

The pH of saliva and urine was chronically higher in patients with *SCN1A*-related epilepsy than in the control group. This is the first study to evaluate the pH of saliva and urine in *SCN1A*-related epilepsy patients, which would be the first step in the application of preventive therapeutic strategies for patients with *SCN1A*-related epilepsy.

## Data availability statement

The data that support the conclusions of this article are available from the corresponding author, upon reasonable request.

## Ethics statement

The studies involving human participants were reviewed and approved by Chinese PLA General Hospital. Written informed consent to participate in this study was provided by the participants' legal guardian/next of kin. Written informed consent was obtained from the minor(s)' legal guardian/next of kin for the publication of any potentially identifiable images or data included in this article.

## Author contributions

QL and L-PZ participated in the study design, data analysis, and manuscript drafting. Y-YW contributed to the data analysis and manuscript drafting. H-MC, Q-HW, and X-YY conducted data collection. All authors have read and approved the final manuscript.

## Conflict of interest

The authors declare that the research was conducted in the absence of any commercial or financial relationships that could be construed as a potential conflict of interest.

## Publisher's note

All claims expressed in this article are solely those of the authors and do not necessarily represent those of their affiliated organizations, or those of the publisher, the editors and the reviewers. Any product that may be evaluated in this article, or claim that may be made by its manufacturer, is not guaranteed or endorsed by the publisher.

## Abbreviations

VGSCs: Voltage-gated sodium ion channels
Nav1.1: α1 subunit of the sodium channel
CLB: clobazam
LEV: levetiracetam
ZNS: zonisamide
VPA: sodium valproate
TPM: topiramate
VGB: vigabatrin
ACMG: the American College of Medical Genetics and Genomics
ASIC1a: Acid-sensing ion channel 1a
NMDA: N-methyl-D-Aspartate.
