# Supplementary material for: A rise in saliva and urine pH in children with SCN1A-related epilepsy: An exploratory prospective controlled study
Source: Front Neurol. 2022 Sep 27;13:982050. doi: 10.3389/fneur.2022.982050 (PMC9552845; doi:10.3389/fneur.2022.982050)
Supplement: Supplementary file 1 [file Data_Sheet_1.docx]

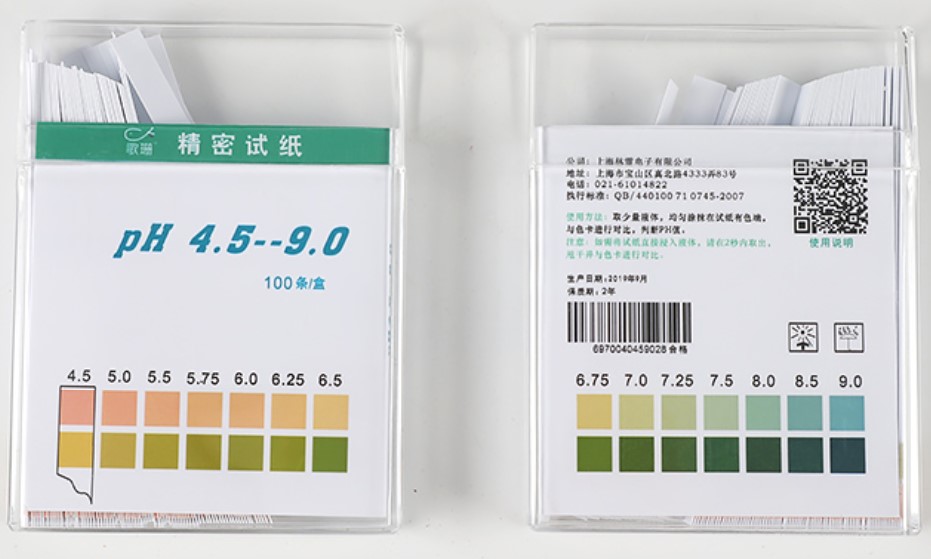


Supplementary Figure 1. pH test paper


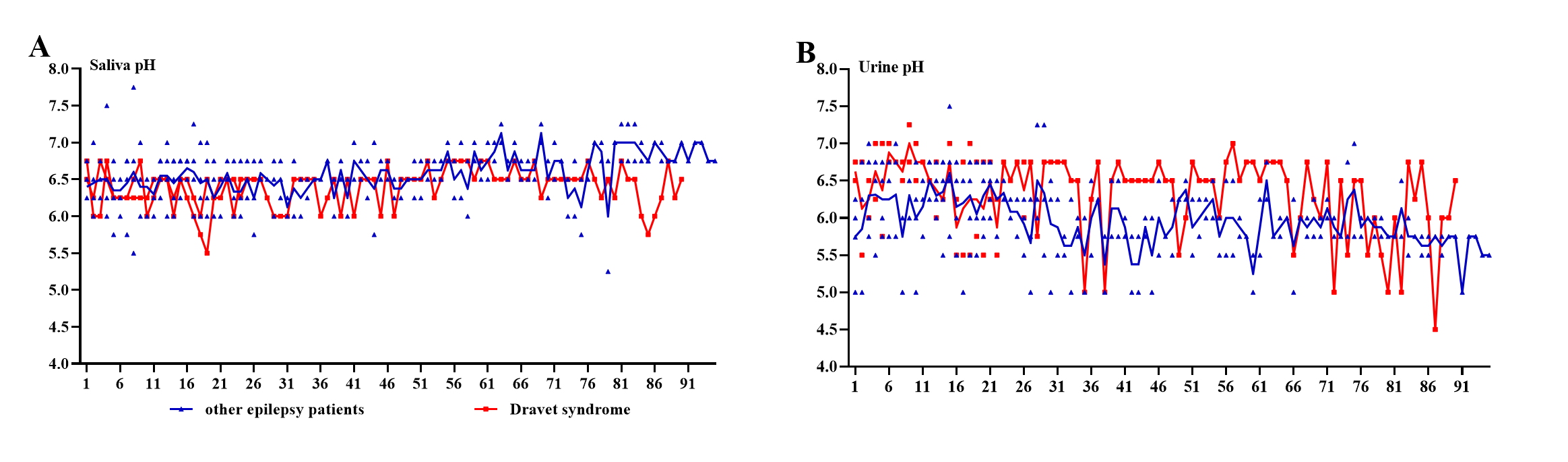


Supplementary Figure 2. The pH of Dravet syndrome patients and other epilepsy patients. Figure 2A shows saliva pH. The saliva pH of Dravet syndrome patients was lower than that of other epilepsy patients. Figure 2B shows urine pH, which distribution of Dravet syndrome patients was higher than that in the other epilepsy patients. The horizontal axis is the number of monitoring days, the vertical axis is the pH, and the curve is the average pH. The blue triangle is the pH of other epilepsy patients, and the red square is the pH of Dravet syndrome patients.


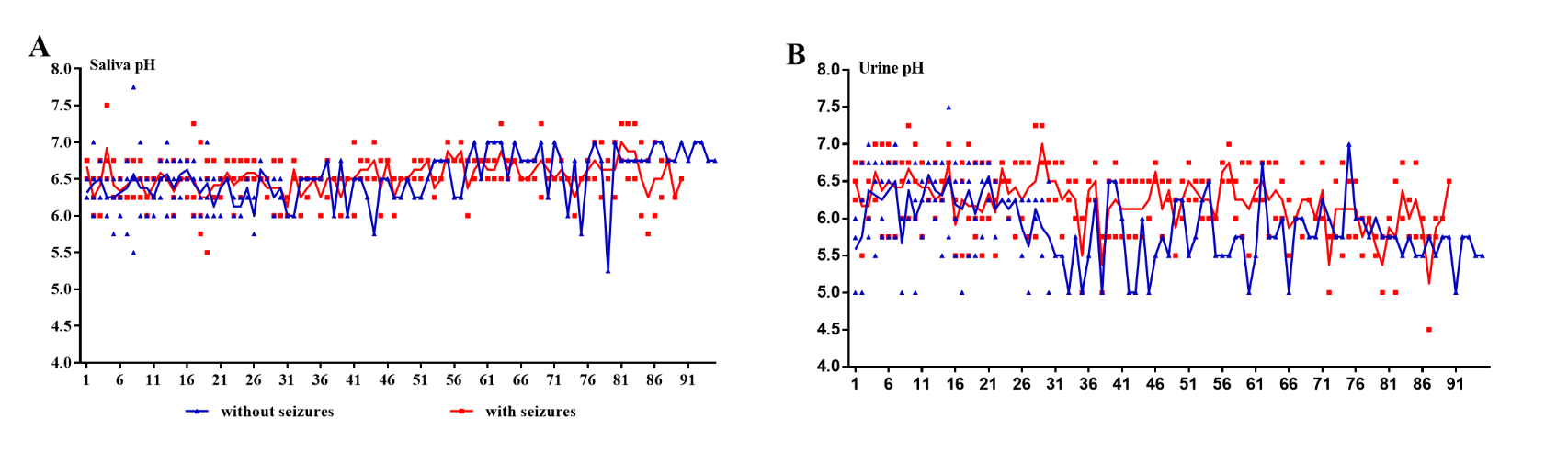


Supplementary Figure 3. The pH of patients who had seizures or not. Figure 3A shows saliva pH. There was no difference in saliva pH between the patients who had seizures or not. Figure 3B shows urine pH, which distribution of patients with seizures was higher than that in the patients without seizures. The horizontal axis is the number of monitoring days, the vertical axis is the pH, and the curve is the average pH. The blue triangle is the pH of patients without seizures, and the red square is the pH of patients with seizures.
